# Gadd45a opens up the promoter regions of miR-295 facilitating pluripotency induction

**DOI:** 10.1038/cddis.2017.497

**Published:** 2017-10-12

**Authors:** Linpeng Li, Keshi Chen, Yi Wu, Qi Long, Danyun Zhao, Bochao Ma, Duanqing Pei, Xingguo Liu

**Affiliations:** 1 CAS Key Laboratory of Regenerative Biology, Joint School of Life Sciences, Guangzhou Institutes of Biomedicine and Health, Chinese Academy of Sciences, Guangzhou, Guangzhou Medical University, Guangzhou, China; 2 Guangdong Provincial Key Laboratory of Stem Cell and Regenerative Medicine, South China Institute for Stem Cell Biology and Regenerative Medicine, Guangzhou Institutes of Biomedicine and Health, University of Chinese Academy of Sciences, Chinese Academy of Sciences, Guangzhou, China

## Abstract

MicroRNAs (miRNAs) play crucial roles in the establishment of pluripotent state by controlling pluripotent network. However, the molecular mechanisms controlling miRNAs during somatic cell reprogramming remain obscure. In this study, we show Gadd45a (growth arrest and DNA-damage-inducible protein 45a) enhances reprogramming by activating miR-295. Furthermore, we show that Gadd45a binds the promoter regions of miR-295. Nuclease accessibility assay indicates that Gadd45a opens the promoter regions of miR-295. Levels of H3K9Ac and H3K27Ac on the promoter regions of miR-295 were also increased. In conclusion, our results indicate that Gadd45a relaxes the promoter regions of miR-295 and promotes the expression of miR-295 during reprogramming, implying a concise mechanism of Gadd45a and miR-290 cluster cooperation in cell-fate determination.

MicroRNAs (miRNAs) are endogenous short noncoding RNAs that regulate gene expression and control many physiological processes including development, homeostasis and metabolism.^1, 2^ Aberrant miRNA expression is involved in cancer and many other diseases.^3, 4^ Recent studies have revealed that miRNAs play critical roles in pluripotency maintenance and somatic cell reprogramming.^5, 6^

Several miRNAs including miR-291-3p, miR-294 and miR-295 form the miR-290 cluster and belong to the embryonic stem cell-specific cell cycle regulating miRNAs^7^ that enhance the generation of mouse induced pluripotent stem cells (iPSCs) using Sox2, Klf4 and Oct4 (SKO).^8^ The miR-290 cluster is involved in the epigenetic control of gene expression and is suggested to promote the G1/S transition to accelerate cell proliferation.^7, 9^ Other miRNAs, such as the miR-302 and miR-200 clusters, have also been shown to enhance reprogramming through promoting the mesenchymal-to-epithelial transition,^10, 11, 12^ an early event in the reprogramming of fibroblasts.^13, 14^

Several groups have reported that iPSCs could be generated using only miRNAs.^15, 16^ The first such study employed the miR-302 cluster, whereas others used miR-200c, miR-302s and miR-369s.^15, 16^ There are also miRNA families such as miR-34, miR-21 and miR-29a that interfere with reprogramming.^17, 18^ miR-34 is a target of P53, a known repressor of reprogramming.^17, 19, 20^ miR-21 and miR-29a are abundantly expressed in mouse embryonic fibroblasts (MEFs) and depletion of each results in enhanced reprogramming efficiency.^18, 21^

Gadd45 (growth arrest and DNA-damage-inducible protein 45a) is a potential RNA-binding protein involved in many cellular processes including cell cycle, senescence, tumor progression, DNA repair and active DNA demethylation.^22, 23, 24^ The expression of Gadd45a has been detected in many mammalian cells, and the rapid induction of Gadd45a upon environmental stress and drug therapies has been observed in every cell type tested to date, which is a protective mechanism against DNA damage.^24, 25^ Mechanistically, Gadd45a interacting with core histones and opening chromatin is important for DNA damage repair.^24, 26^ The expression of Gadd45a was also analyzed during embryonic development. Section and whole mouse embryos analysis shows most prominent Gadd45a expression in the tip of the closing neural tube, the cranial and dorsal root ganglia and the somites that predominantly encompass areas of cell differentiation.^27^

Previously, we reported that Gadd45a is a heterochromatin relaxer that enhances iPSC generation by promoting pluripotent gene expression during reprogramming.^26^ Here, we demonstrate that Gadd45a relaxes the promoter regions of miR-295 and increases the expression of miR-295 during reprogramming, showing a novel concise targeting of Gadd45a in noncoding RNA in cell-fate determination.

## Results

### Gadd45a upregulates miR-290 cluster in reprogramming

**Figure 1:**
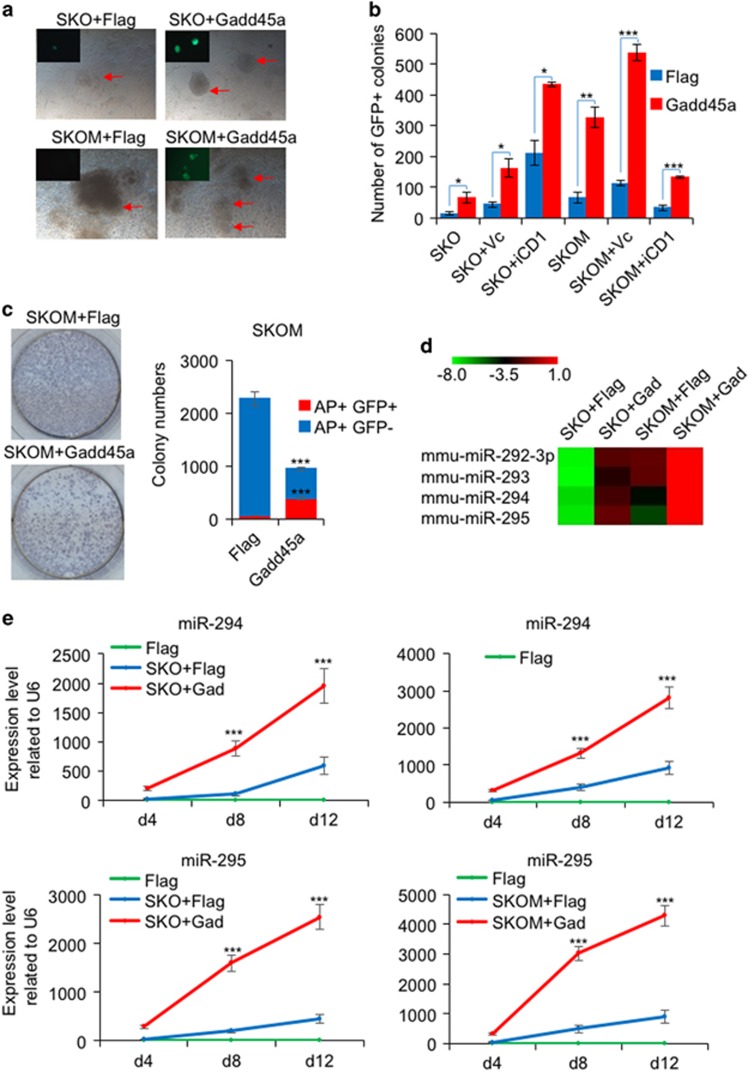
Gadd45a upregulates miR-290 clusters in reprogramming. (**a**) Phase contrast and fluorescence photographs of MEFs infected with SKO plus Flag or Gadd45a, SKOM plus Flag or Gadd45a. Flag is the empty vector control. Arrows point to emerging colonies. (**b**) Gadd45a significantly enhances iPSC generation in various mediums. In the absence of vitamin C, the numbers of GFP+ colonies were counted at day 17; in the presence of vitamin C, the numbers of GFP+ colonies were counted at day 13; in iCD1 medium, the numbers of GFP+ colonies were counted at day 7. Data are represented as mean±S.D.; *n*=3. *P≤0.05, **P≤0.01, ***P≤0.001. (**c**) Gadd45a overexpression blocks the formation of partially reprogrammed cells. The colonies were stained for alkaline phosphatase (AP). The numbers of AP+GFP+ colonies and AP+GFP− colonies were counted. Data are represented as mean±S.D. *n*=3; ***P≤0.001. (**d**) Heatmaps depicting the relative fold change of miRNA expression at day 8 post infection by miRNA microarray. Red and green colors indicated increased and decreased expression, respectively. (**e**) qPCR analysis of miR-294 and miR-295 expression level during SKO- and SKOM-mediated reprogramming in the presence and absence of Gadd45a. Data are represented as mean±S.D.; *n*=3. ***P≤0.001

Previously, we reported that Gadd45a is a heterochromatin relaxer that could enhance somatic cell reprogramming efficiency.^26^ By counting the GFP+ colony number and FACS quantification of GFP+ cells, we confirmed that Gadd45a overexpression significantly promotes the appearance of GFP+ cells in MEFs carrying a transgenic *Oct4*-GFP promoter infected with SKO or SKO plus c-Myc (SKOM) (Figures 1a and b and Supplementary Figures S1a and b). The iPSC colonies generated by SKO plus Gadd45a have normal karyotypes and expression of endogenous pluripotency markers such as Rex1, Nanog and Ssea1 (Supplementary Figures S1c–e). These iPSCs were able to form chimeric mice with germline transmission (Supplementary Figure S1f). We further showed that in the presence of vitamin C^28^ or in a high efficiency medium iCD1,^29^ Gadd45a expression produces many more iPSCs in both SKO- and SKOM-mediated reprogramming (Figure 1b). During SKOM-mediated reprogramming, there are alkaline phosphatase (AP) expression and GFP-negative (AP+GFP−) colonies considered partially reprogrammed cells.^28, 30^ We counted the AP+ colonies and found a high ratio of AP+GFP+ to AP+GFP− colonies upon Gadd45a overexpression (Figure 1c). These results demonstrate that Gadd45a can enhance reprogramming in various media and suppress the formation of partially reprogrammed cells.

To investigate the miRNAs regulated by Gadd45a during reprogramming, we performed miRNA microarray in both SKO- and SKOM-mediated reprogramming at day 8. Gadd45a was found to regulate 23 miRNAs in SKO-mediated reprogramming and 45 miRNAs in SKOM-mediated reprogramming (Supplementary Figure S2). Among them, four miRNAs were found to be upregulated in both the SKO and SKOM data sets: miR-292-3p, miR-293, miR-294 and miR-295, all of which belong to the miR-290 cluster (Figure 1d). In addition there was one miRNA, miR-324-3p, that was found to be downregulated by Gadd45a expression in both SKO and SKOM reprogramming (Supplementary Figure S2).

To substantiate this finding, we evaluated the expression of the five miRNAs by qPCR at different days during reprogramming. Indeed, miR-292-3p, miR-293, miR-294 and miR-295 were significantly upregulated by Gadd45a during both SKO- and SKOM-mediated reprogramming (Figure 1e and Supplementary Figure S3a). However, Gadd45a did not downregulate miR-324-3p during SKO reprogramming, and in SKOM reprogramming, Gadd45a inhibited the expression of miR-324-3p only at day 8 (Supplementary Figure S3a). Notably, Gadd45a alone failed to up-regulate the expression of miR-290 cluster or downregulate the expression of miR-324-3p (Supplementary Figure S3b). Furthermore, we found Gadd45a did not alter the cell cycle of MEFs after overexpression (Supplementary Figure S3c). Together, it appears that Gadd45a promotes reprogramming by increasing miR-290 cluster.

### Gadd45a and miR-295 function in the same way to enhance reprogramming

**Figure 2:**
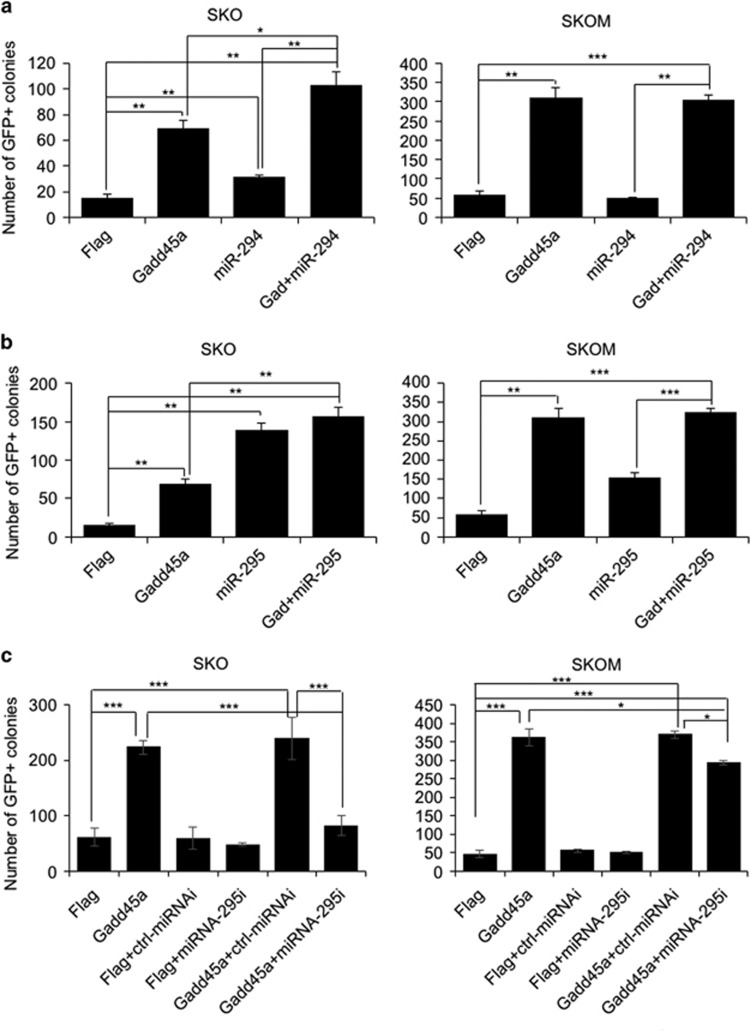
Gadd45a enhances reprogramming through activating miR-295. (**a**) Efficiencies of SKO- and SKOM-mediated reprogramming were tested in the presence of Gadd45a and miR-294. The numbers of GFP+ colonies were counted at day 17. Data are represented as mean±S.D. *n*=3.; *P≤0.05, **P≤0.01, ***P≤0.001. (**b**) Efficiencies of SKO- and SKOM-mediated reprogramming were tested in the presence of Gadd45a and miR-295. The numbers of GFP+ colonies were counted at day 17. Data are represented as mean±S.D.; *n*=3. **P≤0.01, ***P≤0.001. (**c**) Efficiencies of SKO- and SKOM-mediated reprogramming were tested in the presence of Gadd45a and miR-295 inhibitor. The numbers of GFP+ colonies were counted at day 22. Data are represented as mean±S.D.; *n*=3. *P≤0.05, ***P≤0.001

Among the four upregulated miRNAs, miR-294 and miR-295 are embryonic stem cell-specific miRNAs and have already been reported to enhance SKO- but not SKOM-mediated reprogramming.^8^ We introduced miR-294 or miR-295 to SKO- and SKOM-mediated reprogramming in the presence and absence of Gadd45a (Supplementary Figure S4a). As reported, miR-294 promoted iPSC generation with SKO but not SKOM (Figure 2a). In SKO-mediated reprogramming, miR-294 further increased the reprogramming efficiency in the presence of Gadd45a (Figure 2a). We found, however, that miR-295 enhanced both SKO- and SKOM-mediated reprogramming (Figure 2b and Supplementary Figure S4b). In SKO-mediated reprogramming, miR-295 generated more iPSCs than Gadd45a alone, whereas in SKOM-mediated reprogramming, miR-295 was less efficient than Gadd45a (Figure 2b). Combining miR-295 and Gadd45a failed to further enhance reprogramming efficiency in both SKO- and SKOM-mediated reprogramming beyond the levels achieved by either miR-295 or Gadd45a alone, respectively (Figure 2b).

To further investigate whether miR-294 or miR-295 is required in Gadd45a enhancing reprogramming, we tried the inhibitors of miR-294 and miR-295 in reprogramming (Supplementary Figure S4c). Chemically modified, single-stranded nucleic acids were designed as miRNA inhibitors to specifically bind to and inhibit endogenous miRNAs. Inhibition of endogenous miR-294 has no effect on reprogramming, neither in the absence nor in the presence of Gadd45a (Supplementary Figure S4d). In addition, the inhibitor of miR-295 has no obvious effect on reprogramming in the absence of Gadd45a (Figure 2c). However, in the presence of Gadd45a, the inhibitor of miR-295 inhibits both SKO- and SKOM-mediated reprogramming significantly (Figure 2c). These results suggest that Gadd45a is enhancing reprogramming through activating miR-295. As the inhibition of miR-295 did not completely impair the increased reprogramming efficiency by Gadd45a overexpression, extra mechanisms may act downstream of Gadd45a to mediate the enhancement of reprogramming, such as activating endogenous pluripotency genes reported previously.

**Figure 3:**
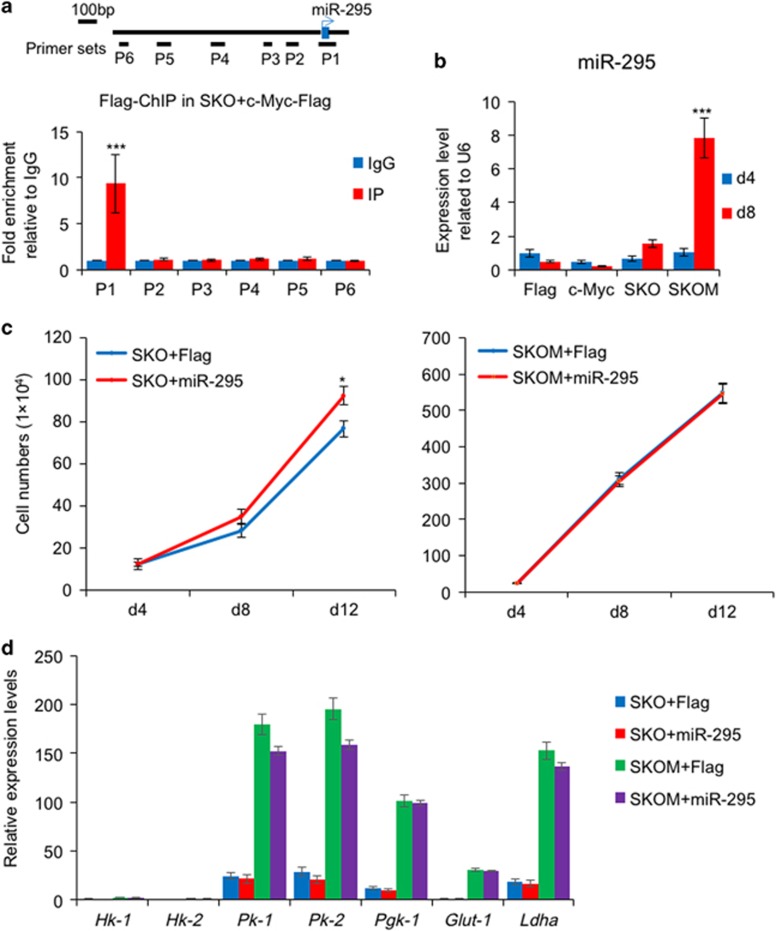
Characterization of the relationship between c-Myc and miR-295. (**a**) ChIP-PCR analysis shows that c-Myc binds to the promoter of miR-295. Data are represented as mean±S.D.; *n*=3. ***P≤0.001. (**b**) qPCR analysis of miR-295 expression level in MEFs infected with Flag, c-Myc, SKO and SKOM at days 4 and 8. Data are represented as mean±S.D.; *n*=3. ***P≤0.001. (**c**) MEFs infected with SKO plus Flag and miR-295, SKOM plus Flag and miR-295 were plated in 12-well plate, and were counted at different days. Data are represented as mean±S.D.; *n*=3. *P≤0.05. (**d**) qPCR analysis of several glycolysis genes expression level in MEFs infected with SKO plus Flag and miR-295, SKOM plus Flag and miR-295. Data are represented as mean±S.D.; *n*=3

It is noted that there is a reduction impact on reprogramming efficiency by miR-295 in the presence of SKOM compared with SKO (Figure 2b). It has also been reported that Myc proteins could bind to the promoter region of the miR-290 cluster.^8^ To explore the relationship between c-Myc and miR-295, we performed chromatin immunoprecipitation PCR assays (ChIP-PCR) and showed c-Myc binds to the proximal promoter region of miR-295 (Figure 3a). Then, we detected the expression of miR-295 induced by factors, and found that miR-295 was robustly activated at day 8 in SKOM-mediated reprogramming, whereas single c-Myc or SKO could not (Figure 3b). These results indicate that miR-295 acts downstream of c-Myc in SKOM-mediated reprogramming.

As c-Myc has been reported to induce gain of proliferation and metabolic changes from oxidative metabolism to glycolysis as the first wave in reprogramming,^31^ we then asked whether miR-295 is involved in this function of c-Myc. We quantified the proliferation of MEFs in reprogramming and found that miR-295 could promote cell proliferation at the late phase of SKO-mediated reprogramming but to a much less extent of c-Myc, and had no effect during the whole process of SKOM-mediated reprogramming (Figure 3c). Next, we detected the expression of glycolysis genes, and showed that unlike c-Myc, miR-295 did not increase the expression of glycolysis genes (Figure 3d). These results indicate that miRNA-295 is not involved in c-Myc function in deriving cell proliferation and metabolic transition.

### Gadd45a binds to and opens up the promoter regions of miR-295

**Figure 4:**
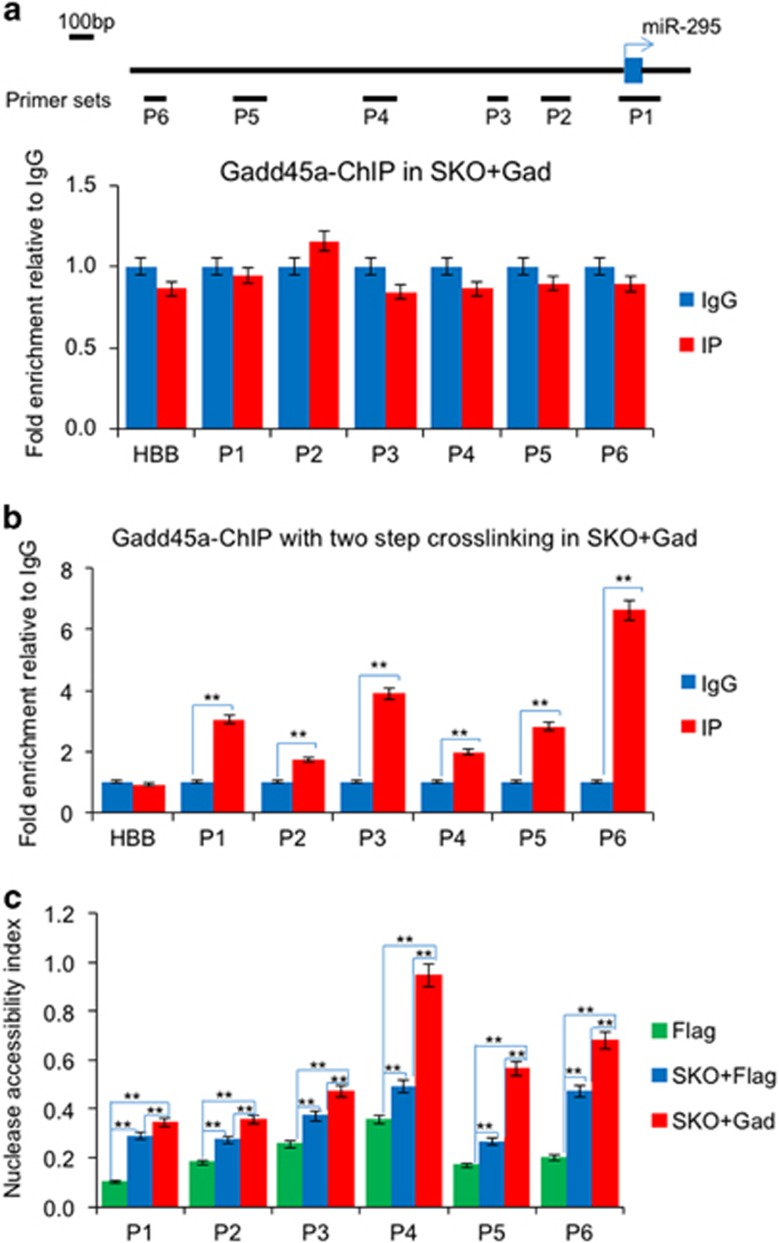
Gadd45a acts on the promoter regions of miR-295 and relaxes the promoter regions of miR-295. (**a**) ChIP-PCR analysis shows that Gadd45a does not bind directly to the promoter of miR-295. HBB was used as negative control. (**b**) ChIP-PCR analysis with DSG crosslinking demonstrates that Gadd45a interacts with chromatin in the promoter regions of miR-295. HBB was used as negative control. Data are represented as mean±S.D.; *n*=3. **P≤0.01. (**c**) The chromatin compaction of the indicated regions was detected by a nuclease accessibility assay. Genomic DNA was purified from MEFs infected with Flag alone and SKO plus Flag or Gadd45a on day 8. Data are represented as mean±S.D.; *n*=3. **P≤0.01

To gain further insight into how Gadd45a regulates miR-295 expression in reprogramming, we designed several primers in the promoter regions of miR-295 and performed ChIP-PCR (Figure 4a). Using this technique we found no interactions between Gadd45a and miR-295 promoter regions (Figure 4a). It was reported that Gadd45a interacts with core histones,^24, 26^ and hence we performed ChIP using a two-step crosslinking, first crosslinking with disuccinimidyl glutarate before ChIP-PCR. Using this technique, we found that Gadd45a could bind to all of the promoter regions of miR-295 (Figure 4b).

We showed previously that Gadd45a is a heterochromatin relaxer that operates on the promoter regions of pluripotent genes in reprogramming.^26^ We detected chromatin compaction status in miR-295 promoter regions by a nuclease accessibility assay^32^ that was verified using positive and negative controls (Supplementary Figure S5). The tested regions displayed more open structures upon infection with SKO compared with MEFs infected with the Flag control (Figure 4c). Gadd45a was able to further increase the nuclease access in SKO reprogramming (Figure 4c).

These results were consistent with our previous report that Gadd45a is a heterochromatin relaxer and further demonstrated Gadd45a opens the promoter regions of miR-295.

### Gadd45a increases the levels of H3K9Ac and H3K27Ac

**Figure 5:**
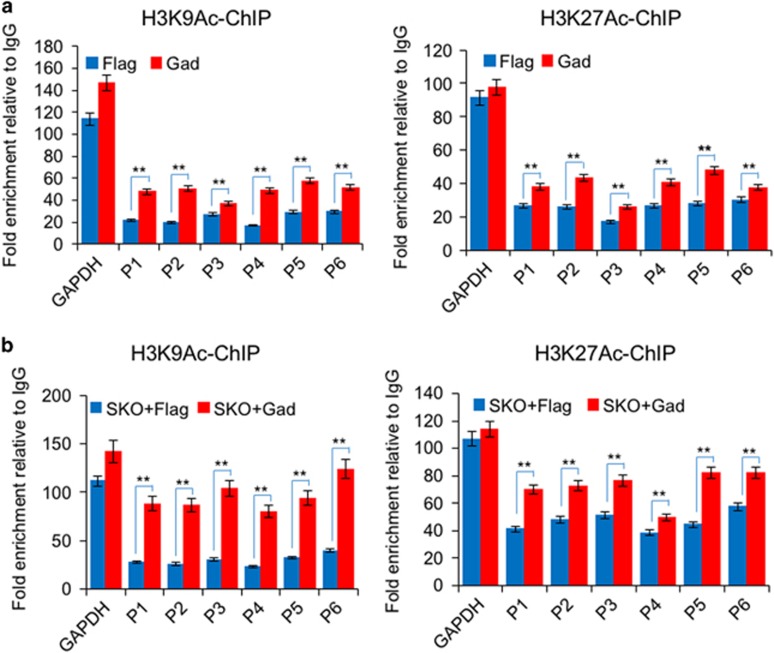
Gadd45a increases the histone acetylation level in reprogramming. (**a**) ChIP-PCR analysis of H3K9Ac and H3K27Ac modification levels in the indicated regions of MEFs infected with Flag or Gadd45a. Data are represented as mean±S.D.; *n*=3. **P≤0.01. (**b**) ChIP-PCR analysis of H3K9Ac and H3K27Ac levels in the indicated regions of MEFs infected with SKO plus Flag or Gadd45a. Data are represented as mean±S.D.; *n*=3. **P≤0.01

**Figure 6:**
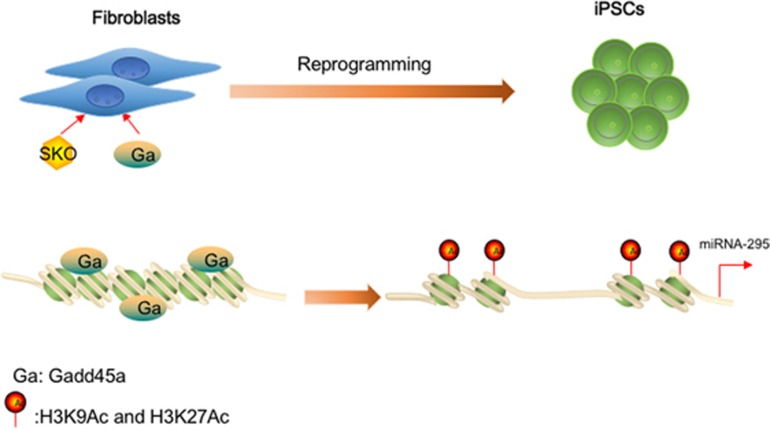
Schematic model of how Gadd45a activates miR-295 during reprogramming. Gadd45a activates miR-295 by relaxing its promoter regions during reprogramming

Histone acetylation is a marker of open chromatin and is associated with active gene promoters and transcription.^33, 34^ We performed ChIP assays with antibodies targeting histone H3 lysine 9 acetylation (H3K9Ac) and histone H3 lysine 27 acetylation (H3K27Ac) at the promoter regions of miR-295. Overexpression of Gadd45a increased H3K9Ac and H3K27Ac levels (Figure 5a). In SKO-mediated reprogramming, the ChIP assays also showed an increase of the H3K9Ac and H3K27Ac levels in the presence of Gadd45a (Figure 5b). The increase of histone acetylation levels at the promoter regions of miR-295 is in agreement with the activation of miR-295 expression in the presence of Gadd45a during reprogramming (Figure 6).

## Discussion

In this report, we show that Gadd45a promotes the expression of miR-290 cluster miRNAs during reprogramming. Further analysis indicated that Gadd45a activates miR-295 to enhance reprogramming. Besides, in human cells, the orthologs of the mouse miR-290 cluster are miR-371-373 cluster.^11^ Our unpublished data show that miR-372-5p, which was reported to promote the human fibroblast reprogramming,^11^ could be upregulated upon GADD45A overexpression in SKOM-mediated human skin fibroblast reprogramming, and further work needs to be done.

The miRNAs are essential in both embryonic development and somatic cell reprogramming.^35, 36^ Mouse knockouts for the RNA-processing proteins Dicer1 and Dgcr8 result in global defects of miRNA maturation and are lethal during embryogenesis.^36^ Dgcr8 knockout mouse embryonic stem cells (mESCs) display defects in proper differentiation and fibroblasts lacking mature miRNAs are not able to generate iPSCs, suggesting that miRNAs play crucial roles in cell-fate determination.^35, 37^

Analysis of the putative target genes of miRNAs can provide insight into the mechanisms of reprogramming. Many studies have revealed that miRNAs involved in cell cycle, mesenchymal-to-epithelial transition, DNA methylation and apoptosis can be used to enhance or inhibit pluripotency induction.^5, 6, 21^ For example, the miR-290 cluster is highly expressed in mESCs and inhibits several key regulators of the G1/S transition, thus accelerating cell proliferation by promoting the G1/S transition.^7^ The miR-290 cluster targets and silences Rbl2, a transcriptional repressor of DNA methyltransferases 3a and 3b, to regulate DNA methylation in ESCs.^9, 38^ The miR-290 cluster was also identified to directly target caspase 2 and etoposide-induced 2.4 mRNA, a Tp53 target, thus linking the survival of mESCs with DNA damage pathways.^39^ Our unpublished data indicate that the expression of miR-295 shows no difference between Gadd45a overexpression, Gadd45a knockout and wild-type ESCs. These suggest that our finding of Gadd45a activating miR-295 may play its role in cell transition such as pluripotency acquirement or exit but not pluripotency maintaining.

Previously, we found that Gadd45a could relax the promoter regions of pluripotency genes and promote their expression during reprogramming.^26^ In this study, we showed that Gadd45a likewise promotes the expression of miR-295, providing further evidences for the function of Gadd45a in chromatin relaxation. Another group reported that Gadd45a could be recruited to the promoter of rRNA genes, resulting in increased pre-rRNA synthesis.^40^ Our results demonstrate that Gadd45a regulates the expression of genes and noncoding RNAs by acting on chromatin. It will be interesting to test whether Gadd45a also regulates other noncoding RNAs such as lncRNAs, piwiRNAs. Moreover, considering Gadd45a expression has been detected in the closing neural tube, the ganglia and the somites during mammalian embryonic development,^27^ and upon environmental stress and drug therapies in the cellular response,^24, 25^ our finding of Gadd45a activating miR-295 by chromatin relaxation would shed light on the studies of Gadd45a functions. Indeed, Gadd45a has been reported to play role in differentiation,^26, 41^ and in future it would be interesting to test the possible role of Gadd45a-miR-295 in these processes.

Gadd45a was early identified as an RNA binding protein, but which RNAs bound to Gadd45a in a physiological context remained unknown.^42^ We and others have found that Gadd45a regulates the expression of several noncoding RNAs.^40^ These findings suggest a complex connection between Gadd45a and RNAs in which Gadd45a may cooperate with RNAs to regulate physiological processes. For instance, Gadd45a could induce G2/M arrest whereas the miR-290 cluster, which is regulated by Gadd45a, promotes the G1/S transition.^7, 43^ Moreover, Gadd45a has been reported to mediate active DNA demethylation,^23^ whereas the miR-290 cluster is involved in *de novo* DNA methylation.^9^ Altogether, these studies suggest that Gadd45a cooperates with the miR-290 cluster in regulating the cell cycle and DNA methylation status to maintain cell homeostasis and determine cell fate.

## Materials and methods

### Cell lines

OG2-MEFs were derived from E13.5 embryos obtained by crossing OG2 male mice (a transgenic *Oct4* promoter driving GFP expression) with 129/sv female mice and used for reprogramming within two passages. MEFs and plat-E cells were maintained in DMEM/high glucose supplemented with 10% fetal bovine serum (FBS) (Hyclone, Logan, UT, USA). Mouse iPSCs and ESCs were maintained in a media containing DMEM/high glucose (Hyclone)+N2 (Gibco, Waltham, MA, USA)+B27 (Gibco)+NEAA (Gibco)+GlutaMax (Gibco)+CHIR99021 (Selleck, Houston, TX, USA)+PD0325901 (Selleck)+lif. The cells were obtained with approval from the ethics committee of the Guangzhou Institutes of Biomedicine and Health, Chinese Academy of Sciences (Guangzhou, China).

### Plasmid construction

All expression vectors were based on the retroviral pMXs backbone and purchased from Addgene (Cambridge, MA, USA). The miRNAs were amplified from genomic DNA of MEFs and cloned into the pMXs vector. Primers for miR-294 were: forward (5′-GTGTTGCATCATTTGGGTGTCATCTGTGG-3′) and reverse (5′-TCATCCCAGTTCCAGGAAACCTTCATCTGGATTC-3′).

Primers for miR-295 were: forward (5′-TTATCCTTTTGGCATTGAATAATCCTTAATCTTCAG-3′) and reverse (5′-TCAAAACAAAAACTGAGAAAGCAGCAAAATCAAGAG-3′).

### Virus infection and iPSC generation

Retroviral vectors (pMXs) carrying the factors were transfected into plat-E cells using PEI (Polyscience, Niles, IL, USA) transfection. The viral supernatants were collected 48 and 72 h after transfection and filtered before infecting MEFs with polybrene (Sigma, St. Louis, MO, USA).

Oct4, Sox2, Klf4, c-Myc and other plasmids were transfected into plat-E cells using PEI (Polyscience) to generate viral stocks that infect OG2-MEFs cultured in medium containing 15% FBS (Gibco)+NEAA (Gibco)+GlutaMax (Gibco)+sodium pyruvate (Gibco)+*β*-mercaptoethanol (Invitrogen, Carlsbad, CA, USA)+lif. Vitamin C (Sigma) was added at 50 *μ*g ml^−1^. iCD1 medium was obtained from Dr. Jiekai Chen’s lab (Guangzhou Institutes of Biomedicine and Health, CAS). The GFP+ colonies were counted at the indicated days. iPSC colonies were picked up and characterized. Chimeras were generated by injecting iPSCs into blastocysts derived from ICR mice, followed by implantation into pseudopregnant ICR mice. F2 mice were breeding with chimeric mice and ICR mice to determine germline transmission of iPSCs.

The miRNA inhibitors were bought from GeneCopoeia (Rockville, MD, USA) and transfected into MEFs with PEI following the manufacturer’s instructions.

### AP staining

After washing with PBS, cells were fixed with 4% paraformaldehyde, incubated at room temperature for 8 min and washed twice with 1 ml PBS. Freshly prepared AP staining solution (4.5 *μ*l 50 mg ml^−1^ nitro blue tetrazolium, 3.5 *μ*l 50 mg ml^−1^ 5-bromo-4-chloro-3-indolyl phosphate in 100 mM Tris-HCl, pH 9.5, 100 mM NaCl, 50 mM MgCl_2_) was added to the cells before incubating in dark at room temperature for 10 min. The staining solution was aspirated, and plates were washed with PBS and imaged.

### miRNA microarray

The miRNA mircroarrays were performed using Agilent (Santa Clara, CA, USA) mouse miRNA (8 × 60K) V16.0 chip. Data were extracted by Feature Extraction software 10.7 (Agilent Technologies, Santa Clara, CA, USA) with default settings. Raw data were normalized by the Quantile algorithm, Gene Spring Software 11.0 (Agilent Technologies). The experiment and data analysis were performed by Shanghai Biotechnology (Shanghai, China). The miRNAs showing significant expression changes (FC >2) upon overexpression of Gadd45a were selected and further analyzed.

### Accession number

The accession number for the miRNA microarrays gathered in this study is GSE56942.

### mRNA extraction and qPCR

Total RNA was extracted with TRIzol (Invitrogen) and 5 *μ*g RNA was used to generate complementary DNA. The expression levels of pluripotency genes were determined using SYBR Green (Bio-Rad, Hercules, CA, USA) and analyzed with CFX96 Real-Time System (Bio-Rad).

Primers for *Oct4* were: forward: (5′-AGAGGATCACCTTGGGGTACA-3′) and reverse (5′-CGAAGCGACAGATGGTGGTC-3′).

Primers for *Rex1* were: forward: (5′-CCCTCGACAGACTGACCCTAA-3′) and reverse (5′-TCGGGGCTAATCTCACTTTCAT-3′).

Primers for *Nanog* were: forward: (5′-CTCAAGTCCTGAGGCTGACA-3′) and reverse (5′-TGAAACCTGTCCTTGAGTGC-3′).

Primers for *Hk-1* were: forward: (5′-TGGACGACATCAGAACAGAC-3′) and reverse (5′-GGACGATCTCACCCAGGTAC-3′).

Primers for *Hk-2* were: forward: (5′-GCATCATTGTGAAGGAGGTG-3′) and reverse (5′-GTTGTCCAGTCCACGGTTCT-3′).

Primers for *Pk-1* were: forward: (5′-GGCAGGAGTGCTCACCAAGT-3′) and reverse (5′-AGATGCCACGGTACAGATGG-3′).

Primers for *Pk-2* were: forward: (5′-ATCTGTACCGTGGCATCTTC-3′) and reverse (5′-CACAATGACCACATCTCCCT-3′).

Primers for *Pgk-1* were: forward: (5′-GGGGTATTTGAATGGGAAGC-3′) and reverse (5′-CACAGCAAGTGGCAGTGTCT-3′).

Primers for *Ldha* were: forward: (5′-CACAAGCAGGTGGTGGACAG-3′) and reverse (5′-CCCGCCTAAGGTTCTTCATT-3′).

Primers for *Glut-1* were: forward: (5′-GTGGTGTCGCTGTTTGTTGT-3′) and reverse (5′-GGCCACGATGCTCAGATAGG-3′).

### miRNA extraction and qPCR

Total RNA was extracted from cultured cells using TRIzol. Real-time RT-PCR was performed with the All-in-One miRNA qRT-PCR Detection Kit (GeneCopoeia, Rockville, MD, USA) following the manufacturer’s instructions. Mouse snRNA U6 was used as the reference gene and obtained from GeneCopoeia. The relative expression level for each miRNA was normalized to the level of snRNA U6. Individual samples were run in triplicate. Primers were used as following:

miR-295: 5′-GCGCGCAAAGTGCTACTACTTTT-3′.

miR-294: 5′-GCGCAAAGTGCTTCCCTTTT-3′.

miR-292-3p: 5′-AAAGTGCCGCCAGGTTTTGAG-3′.

miR-293: 5′-GCGCACTCAAACTGTGTGACATT-3′.

miR-324-3p: 5′-CCACTGCCCCAGGTGCTG-3′.

### Cell proliferation assay

MEFs were seeded at a density of 1.5 × 10^4^ cells per 12-well plate and infected with viruses. Cells were trypsinized and counted at days 4, 8 and 12 after infection.

### Chromatin immunoprecipitation

Cells were crosslinked with 1% formaldehyde (Sigma) for 15 min at room temperature and then washed three times with PBS, and then harvested by scraping with a spatula. Cells were lysed in SDS buffer (1% SDS, 50 mM Tris-HCl (pH 8.0), 10 mM EDTA and protease inhibitor cocktail) for 10 min at 4 °C and sheared by sonication. Sheared chromatin was diluted by 10 times with ChIP IP buffer (0.01% SDS, 1% Triton X-100, 2 mM EDTA, 50 mM Tris-HCl (pH 8.0), 150 mM NaCl and protease inhibitor cocktail). Antibodies were linked to Dynabeads with protein A or G (Invitrogen) for >3 h at 4 °C in PBST (PBS supplemented with 0.01% Tween-20). Diluted chromatin was incubated with antibodies overnight at 4 °C. After immunoprecipitation, beads were washed with low-salt wash buffer (0.1% SDS, 1% Triton X-100, 2 mM EDTA, 20 mM Tris-HCl (pH 8.0) and 150 mM NaCl), high-salt wash buffer (0.1% SDS, 1% Triton X-100, 2 mM EDTA, 20 mM Tris-HCl (pH 8.0) and 500 mM NaCl), LiCl wash buffer (0.25 M LiCl, 1% NP-40, 1% deoxycholate, 1 mM EDTA and 10 mM Tris-HCl (pH 8.1)) and TE buffer (10 mM Tris-HCl and 1 mM EDTA (pH 8.0)). DNA was extracted with Chelex-100 and used for analysis. ChIP assays using anti-Gadd45a antibodies (Santa Cruz, Dallas, TX, USA), anti-H3K9Ac (Abcam, Cambridge, UK) and anti-H3K27Ac (Abcam) antibodies were performed on day 3 of SKO-mediated reprogramming. ChIP assays using anti-Flag antibodies (Sigma) were performed on day 8 of SKO plus c-Myc-Flag-mediated reprogramming.

Primers for GAPDH were: forward (5′-CCTTCATTGACCTCAACTACA-3′) and reverse (5′-TAGACTCCACGACATACTCA-3′).

Primers for P1 were: forward (5′-CCAGAGACTCCTTGCTTGCT-3′) and reverse (5′-CCAGGAAACCTTCATCTGGA-3′).

Primers for P2 were: forward (5′-TGCAGTTGGCCTAAGTGTTG-3′) and reverse (5′-TCAAATCTGGGTCACTTCCC-3′).

Primers for P3 were: forward (5′-TACTTGCCTTCAACCTGCCT-3′) and reverse (5′-CAGGGACCTGCTGTGGTAAT-3′).

Primers for P4 were: forward (5′-GTCCAGGGGTGGTGAGTCTA-3′) and reverse (5′-ACAGCCAGGGCTACAGAGAA-3′).

Primers for P5 were: forward (5′-ACTCAAACTGGGGGCTCTTT-3′) and reverse (5′-AATGCAACCCCAGTGAAAAC-3′).

Primers for P6 were: forward (5′-CGGTAACTGAGGTGGTCGTT-3′) and reverse (5′-GATGGCCGCTACATAGGTGT-3′).

Primer for HBB were: forward (5′-GAGTGGCACAGCATCCAGGGAGAAA-3′) and reverse (5′-CCACAGGCCAGAGACAGCAGCCTTC-3′)

To perform ChIP with two-step crosslinking, cells were crosslinked with 2 mM Disuccinimidyl Glutarate (Thermo, Waltham, MA, USA) in PBS supplemented with 1 mM MgCl_2_ for 45 min at room temperature. After washing three times with PBS, cells were crosslinked with 1% formaldehyde and continued for ChIP assay as described above.

### Nuclease accessibility assay

Nuclease accessibility assay was performed with EpiQ Chromatin Analysis Kit (Bio-Rad) following instructions by the manufacturer. MEFs were infected with Flag, SKO or SKO plus Gadd45a, and then divided into two groups, one of which was digested with the EpiQ nuclease. After stopping the reaction with stopping buffer, the genomic DNA was purified and subjected to qPCR. The primers were designed from the miR-295 promoter. The nuclease accessibility index was calculated after normalization to an internal control. Primers for GAPDH were: forward (5′-TGCGACTTCAACAGCAACTC-3′) and reverse (5′-CTTGCTCAGTGTCCTTGCTG-3′).

Primers for HBB and P1 to P6 were the same in the Chromatin immunoprecipitation assay.

### Statistics

Data are represented as mean±S.D. Comparison of values between groups was evaluated by the nonpaired two-tailed Student’s *t*-test. *P*≤0.05 was considered statistically significant.

## References

[bib1] Bartel DP. MicroRNAs: genomics, biogenesis, mechanism, and function. Cell 2004; 116: 281–297. 14744438 10.1016/s0092-8674(04)00045-5

[bib2] Bartel DP. MicroRNAs: target recognition and regulatory functions. Cell 2009; 136: 215–233. 19167326 10.1016/j.cell.2009.01.002 PMC3794896

[bib3] Mendell JT, Olson EN. MicroRNAs in stress signaling and human disease. Cell 2012; 148: 1172–1187. 22424228 10.1016/j.cell.2012.02.005 PMC3308137

[bib4] Emde A, Hornstein E. miRNAs at the interface of cellular stress and disease. EMBO J 2014; 33: 1428–1437. 24867813 10.15252/embj.201488142 PMC4194087

[bib5] Leonardo TR, Schultheisz HL, Loring JF, Laurent LC. The functions of microRNAs in pluripotency and reprogramming. Nat Cell Biol 2012; 14: 1114–1121. 23131918 10.1038/ncb2613 PMC6692081

[bib6] Bao X, Zhu X, Liao B, Benda C, Zhuang Q, Pei D et al. MicroRNAs in somatic cell reprogramming. Curr Opin Cell Biol 2013; 25: 208–214. 23332905 10.1016/j.ceb.2012.12.004

[bib7] Wang Y, Baskerville S, Shenoy A, Babiarz JE, Baehner L, Blelloch R. Embryonic stem cell-specific microRNAs regulate the G1-S transition and promote rapid proliferation. Nat Genet 2008; 40: 1478–1483. 18978791 10.1038/ng.250 PMC2630798

[bib8] Judson RL, Babiarz JE, Venere M, Blelloch R. Embryonic stem cell-specific microRNAs promote induced pluripotency. Nat Biotechnol 2009; 27: 459–461. 19363475 10.1038/nbt.1535 PMC2743930

[bib9] Sinkkonen L, Hugenschmidt T, Berninger P, Gaidatzis D, Mohn F, Artus-Revel CG et al. MicroRNAs control de novo DNA methylation through regulation of transcriptional repressors in mouse embryonic stem cells. Nat Struct Mol Biol 2008; 15: 259–267. 18311153 10.1038/nsmb.1391

[bib10] Liao B, Bao X, Liu L, Feng S, Zovoilis A, Liu W et al. MicroRNA cluster 302-367 enhances somatic cell reprogramming by accelerating a mesenchymal-to-epithelial transition. J Biol Chem 2011; 286: 17359–17364. 21454525 10.1074/jbc.C111.235960 PMC3089577

[bib11] Subramanyam D, Lamouille S, Judson RL, Liu JY, Bucay N, Derynck R et al. Multiple targets of miR-302 and miR-372 promote reprogramming of human fibroblasts to induced pluripotent stem cells. Nat Biotechnol 2011; 29: 443–448. 21490602 10.1038/nbt.1862 PMC3685579

[bib12] Hu X, Zhang L, Mao SQ, Li Z, Chen J, Zhang RR et al. Tet and TDG mediate DNA demethylation essential for mesenchymal-to-epithelial transition in somatic cell reprogramming. Cell Stem Cell 2014; 14: 512–522. 24529596 10.1016/j.stem.2014.01.001

[bib13] Li R, Liang J, Ni S, Zhou T, Qing X, Li H et al. A mesenchymal-to-epithelial transition initiates and is required for the nuclear reprogramming of mouse fibroblasts. Cell Stem Cell 2010; 7: 51–63. 20621050 10.1016/j.stem.2010.04.014

[bib14] Samavarchi-Tehrani P, Golipour A, David L, Sung HK, Beyer TA, Datti A et al. Functional genomics reveals a BMP-driven mesenchymal-to-epithelial transition in the initiation of somatic cell reprogramming. Cell Stem Cell 2010; 7: 64–77. 20621051 10.1016/j.stem.2010.04.015

[bib15] Anokye-Danso F, Trivedi CM, Juhr D, Gupta M, Cui Z, Tian Y et al. Highly efficient miRNA-mediated reprogramming of mouse and human somatic cells to pluripotency. Cell Stem Cell 2011; 8: 376–388. 21474102 10.1016/j.stem.2011.03.001 PMC3090650

[bib16] Miyoshi N, Ishii H, Nagano H, Haraguchi N, Dewi DL, Kano Y et al. Reprogramming of mouse and human cells to pluripotency using mature microRNAs. Cell Stem Cell 2011; 8: 633–638. 21620789 10.1016/j.stem.2011.05.001

[bib17] Choi YJ, Lin CP, Ho JJ, He X, Okada N, Bu P et al. miR-34 miRNAs provide a barrier for somatic cell reprogramming. Nat Cell Biol 2011; 13: 1353–1360. 22020437 10.1038/ncb2366 PMC3541684

[bib18] Yang CS, Li Z, Rana TM. microRNAs modulate iPS cell generation. RNA 2011; 17: 1451–1460. 21693621 10.1261/rna.2664111 PMC3153970

[bib19] Kawamura T, Suzuki J, Wang YV, Menendez S, Morera LB, Raya A et al. Linking the p53 tumour suppressor pathway to somatic cell reprogramming. Nature 2009; 460: 1140–1144. 19668186 10.1038/nature08311 PMC2735889

[bib20] Marion RM, Strati K, Li H, Murga M, Blanco R, Ortega S et al. A p53-mediated DNA damage response limits reprogramming to ensure iPS cell genomic integrity. Nature 2009; 460: 1149–1153. 19668189 10.1038/nature08287 PMC3624089

[bib21] Luningschror P, Hauser S, Kaltschmidt B, Kaltschmidt C. MicroRNAs in pluripotency, reprogramming and cell fate induction. Biochim Biophys Acta 2013; 1833: 1894–1903. 23557785 10.1016/j.bbamcr.2013.03.025

[bib22] Rosemary Siafakas A, Richardson DR. Growth arrest and DNA damage-45 alpha (GADD45alpha). Int J Biochem Cell Biol 2009; 41: 986–989. 18760377 10.1016/j.biocel.2008.06.018

[bib23] Barreto G, Schafer A, Marhold J, Stach D, Swaminathan SK, Handa V et al. Gadd45a promotes epigenetic gene activation by repair-mediated DNA demethylation. Nature 2007; 445: 671–675. 17268471 10.1038/nature05515

[bib24] Carrier F, Georgel PT, Pourquier P, Blake M, Kontny HU, Antinore MJ et al. Gadd45, a p53-responsive stress protein, modifies DNA accessibility on damaged chromatin. Mol Cell Biol 1999; 19: 1673–1685. 10022855 10.1128/mcb.19.3.1673 PMC83961

[bib25] Zhan Q. Gadd45a, a p53- and BRCA1-regulated stress protein, in cellular response to DNA damage. Mutat Res 2005; 569: 11. 10.1016/j.mrfmmm.2004.06.055 15603758

[bib26] Chen K, Long Q, Wang T, Zhao D, Zhou Y, Qi J et al. Gadd45a is a heterochromatin relaxer that enhances iPS cell generation. EMBO Rep 2016; 17: 1641–1656. 27702986 10.15252/embr.201642402 PMC5090707

[bib27] Kaufmann LT, Gierl MS, Niehrs C. Gadd45a, Gadd45b and Gadd45g expression during mouse embryonic development. Gene Expr Patterns 2011; 11: 465–470. 21843656 10.1016/j.gep.2011.07.005

[bib28] Esteban MA, Wang T, Qin B, Yang J, Qin D, Cai J et al. Vitamin C enhances the generation of mouse and human induced pluripotent stem cells. Cell Stem Cell 2010; 6: 71–79. 20036631 10.1016/j.stem.2009.12.001

[bib29] Chen J, Liu J, Chen Y, Yang J, Liu H, Zhao X et al. Rational optimization of reprogramming culture conditions for the generation of induced pluripotent stem cells with ultra-high efficiency and fast kinetics. Cell Res 2011; 21: 884–894. 21445094 10.1038/cr.2011.51 PMC3203703

[bib30] Wu Y, Chen K, Liu X, Huang L, Zhao D, Li L et al. Srebp-1 interacts with c-Myc to enhance somatic cell reprogramming. Stem Cells 2016; 34: 83–92. 26388522 10.1002/stem.2209

[bib31] Polo JM, Anderssen E, Walsh RM, Schwarz BA, Nefzger CM, Lim SM et al. A molecular roadmap of reprogramming somatic cells into iPS cells. Cell 2012; 151: 1617–1632. 23260147 10.1016/j.cell.2012.11.039 PMC3608203

[bib32] Yuan W, Wu T, Fu H, Dai C, Wu H, Liu N et al. Dense chromatin activates Polycomb repressive complex 2 to regulate H3 lysine 27 methylation. Science 2012; 337: 971–975. 22923582 10.1126/science.1225237

[bib33] Grunstein M. Histone acetylation in chromatin structure and transcription. Nature 1997; 389: 349–352. 9311776 10.1038/38664

[bib34] Nishida H, Suzuki T, Kondo S, Miura H, Fujimura Y, Hayashizaki Y. Histone H3 acetylated at lysine 9 in promoter is associated with low nucleosome density in the vicinity of transcription start site in human cell. Chromosome Res 2006; 14: 203–211. 16544193 10.1007/s10577-006-1036-7

[bib35] Kim BM, Thier MC, Oh S, Sherwood R, Kanellopoulou C, Edenhofer F et al. MicroRNAs are indispensable for reprogramming mouse embryonic fibroblasts into induced stem cell-like cells. PLoS ONE 2012; 7: e39239. 22737231 10.1371/journal.pone.0039239 PMC3380844

[bib36] Bernstein E, Kim SY, Carmell MA, Murchison EP, Alcorn H, Li MZ et al. Dicer is essential for mouse development. Nat Genet 2003; 35: 215–217. 14528307 10.1038/ng1253

[bib37] Wang Y, Medvid R, Melton C, Jaenisch R, Blelloch R. DGCR8 is essential for microRNA biogenesis and silencing of embryonic stem cell self-renewal. Nat Genet 2007; 39: 380–385. 17259983 10.1038/ng1969 PMC3008549

[bib38] Benetti R, Gonzalo S, Jaco I, Munoz P, Gonzalez S, Schoeftner S et al. A mammalian microRNA cluster controls DNA methylation and telomere recombination via Rbl2-dependent regulation of DNA methyltransferases. Nat Struct Mol Biol 2008; 15: 268–279. 18311151 10.1038/nsmb.1399 PMC2990406

[bib39] Zheng GX, Ravi A, Calabrese JM, Medeiros LAKirak O, Dennis LM et al. A latent pro-survival function for the mir-290-295 cluster in mouse embryonic stem cells. PLoS Genet 2011; 7: e1002054. 21573140 10.1371/journal.pgen.1002054 PMC3088722

[bib40] Schmitz KM, Schmitt N, Hoffmann-Rohrer U, Schafer A, Grummt I, Mayer C. TAF12 recruits Gadd45a and the nucleotide excision repair complex to the promoter of rRNA genes leading to active DNA demethylation. Mol Cell 2009; 33: 344–353. 19217408 10.1016/j.molcel.2009.01.015

[bib41] Zhang RP, Shao JZ, Xiang LX. GADD45A protein plays an essential role in active DNA demethylation during terminal osteogenic differentiation of adipose-derived mesenchymal stem cells. J Biol Chem 2011; 286: 41083–41094. 21917922 10.1074/jbc.M111.258715 PMC3220515

[bib42] Sytnikova YA, Kubarenko AV, Schafer A, Weber AN, Niehrs C. Gadd45a is an RNA binding protein and is localized in nuclear speckles. PLoS ONE 2011; 6: e14500. 21249130 10.1371/journal.pone.0014500 PMC3017548

[bib43] Jin S, Antinore MJ, Lung FD, Dong X, Zhao H, Fan F et al. The GADD45 inhibition of Cdc2 kinase correlates with GADD45-mediated growth suppression. J Biol Chem 2000; 275: 16602–16608. 10747892 10.1074/jbc.M000284200

