# Supplementary material for: Gadd45a opens up the promoter regions of miR-295 facilitating pluripotency induction
Source: Cell Death Dis. 2017 Oct 12;8(10):e3107–. doi: 10.1038/cddis.2017.497 (PMC5682663; doi:10.1038/cddis.2017.497)
Supplement: Supplementary Information [file cddis2017497x1.pdf]

# Supplementary Materials and methods

## Western blot

Cells were washed with PBS for three times, and proteins of the whole cells were separated using RIPA (Beyotime, China). The expression levels of Gadd45a were analyzed using Western blot with anti-Gadd45a antibodies (Santa Cruz, USA). GAPDH was used as the control with anti-GAPDH (Sigma, USA).

## FACS

Cells were trypsinized, washed once with PBS, and resuspended in suitable volume of PBS. Cells were filtered out before analyzed on cFlow Plus C6 (BD, USA) to quantify the induction of *Oct4*-GFP+ cells.

The cell cycle were analyzed with “Cell Cycle and Apoptosis Analysis Kit” (Beyotime, China) following the manufacturer's instructions. Briefly, cells were harvested with PBS, fixed with 70% ethanol, treated with RNase A, stained with propidium iodide for DNA content, and analyzed on cFlow Plus C6. At least 10 000 cells were collected.

## Immunofluorescence

iPSCs were washed once with PBS, and fixed with 4% PFA for 8 min, then stained with antibody anti-Ssea1 (R&D, USA) and anti-Rex1. A Zeiss LSM 800 confocal microscope was used for detection.

# Supplementary Figure Legends

**Figure S1. Gadd45a enhances somatic cell reprogramming.**

(a) Western blot detection of expression of Gadd45a in MEFs infected with indicated virus.

(b) FACS quantification of GFP+ cells in MEFs infected with SKO plus Flag or Gadd45a, SKOM plus Flag or Gadd45a. Data are represented as mean±SD. n=3. ***p≤0.001.

(c) qPCR analysis of several endogenous pluripotent genes in SKOGad iPSC colonies. Data are represented as mean±SD. n=3.

(d) The protein expression level and location of Rex1 and Ssea1 by immunofluorescence. Scale bar: 50 μm. n≥10.

(e) The SKOGad iPSC colonies have normal karyotypes.

(f) Germline transmission of chimeric mice generated from the SKOGad iPSC colonies. The red * indicated the chimeric mouse. The black arrows indicated germline transmission mice.

**Figure S2. Comparisons of miRNAs during reprogramming in the absence and presence of Gadd45a.**

miRNAs regulated by Gadd45a in SKO (left panel) and SKOM (right panel) mediated reprogramming. The common miRNAs are shown in bold. Heatmaps depict the relative fold change of miRNA expression at 8 days post infection as detected by miRNA microarray. Red and green colors indicate increased and

decreased expression, respectively.

**Figure S3. Gadd45a up-regulates miR-292-3p and miR-293 during reprogramming.**

(a) qPCR analysis of miR-292-3p, miR-293 and miR-324-3p expression level during SKO and SKOM mediated reprogramming in the presence and absence of Gadd45a. Data are represented as mean±SD. n=3. ***p≤0.001.

(b) qPCR analysis of miR-292-3p, miR-293, miR-295, miR-295 and miR-324-3p expression level in MEFs infected with Flag or Gadd45a. Data are represented as mean±SD. n=3.

(c) Cell cycle analysis of MEFs infected with Flag or Gadd45a. Flag is the empty vector control. Quantitative analysis of populations in each phage is shown at the right panel. Data are represented as mean±SD. n=3.

**Figure S4. Inhibition of endogenous miR-294 has no effect on reprogramming neither in the absence nor in the presence of Gadd45a.**

(a) qPCR analysis of miR-295 and miR-294 expression level in MEFs infected with SKO or SKOM plus miRNAs. Data are represented as mean±SD. n=3. ***p ≤0.001.

(b) Phase contrast and fluorescence photographs of MEFs infested with SKO and SKOM plus miR-295. Arrows point to emerging colonies.

(c) qPCR analysis of miR-295 and miR-294 expression level in MEFs infected

with SKO or SKOM in the presence of the miRNAs' inhibitors. Data are represented as mean±SD. n=3. ***p≤0.001.

(d) Efficiencies of SKO and SKOM mediated reprogramming were tested in the presence of Gadd45a and miR-294's inhibitor. The numbers of GFP+ colonies were counted at day 22. Data are represented as mean±SD. n=3. ***p≤0.001.

**Figure S5. Gadd45a relaxes the promoter regions of miR-295.**

The chromatin compaction of GAPDH (positive control) and HBB (negative control) was detected by nuclease accessibility assay. Genomic DNA was purified from MEFs infected with Flag alone or SKO plus Flag or Gadd45a. Data are represented as mean±SD. n=3.

# Figure S1

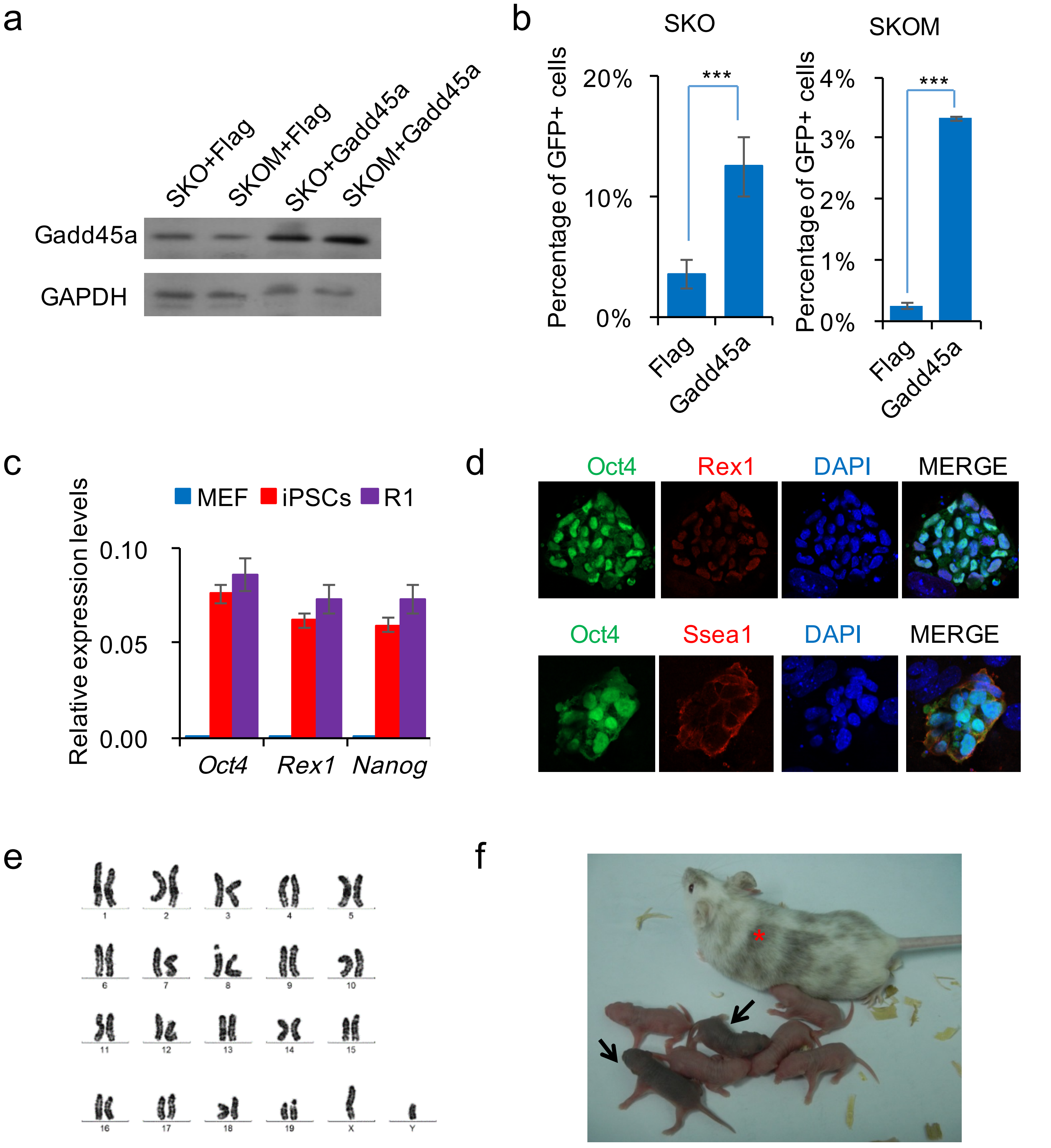
a
SKO+Flag
SKOM+Flag
SKO+Gadd45a
SKOM+Gadd45a
Gadd45a
GAPDH
b
SKO
SKOM
Percentage of GFP+ cells
20%
10%
0%
4%
3%
2%
1%
***
Flag
Gadd45a
c
MEF
iPSCs
R1
Relative expression levels
0.10
0.05
0.00
Oct4
Rex1
Nanog
d
Oct4
Rex1
DAPI
MERGE
Ssea1
e
1
2
3
4
5
6
7
8
9
10
11
12
13
14
15
16
17
18
19
X
Y
f

# Figure S2

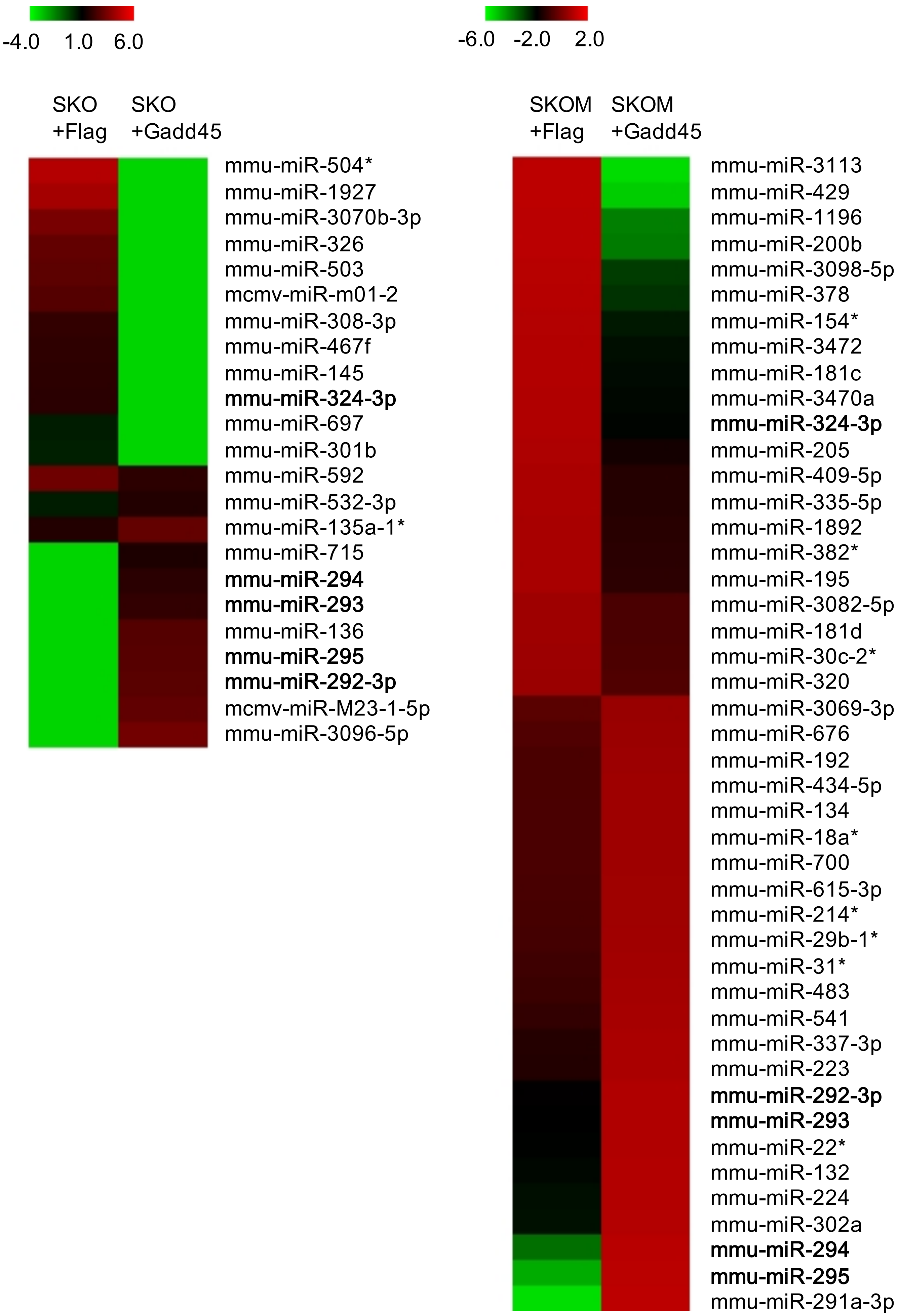
-4.0 1.0 6.0
-6.0 -2.0 2.0
SKO +Flag
SKO +Gadd45
mmu-miR-504*
mmu-miR-1927
mmu-miR-3070b-3p
mmu-miR-326
mmu-miR-503
mcmv-miR-m01-2
mmu-miR-308-3p
mmu-miR-467f
mmu-miR-145
mmu-miR-324-3p
mmu-miR-697
mmu-miR-301b
mmu-miR-592
mmu-miR-532-3p
mmu-miR-135a-1*
mmu-miR-715
mmu-miR-294
mmu-miR-293
mmu-miR-136
mmu-miR-295
mmu-miR-292-3p
mcmv-miR-M23-1-5p
mmu-miR-3096-5p
SKOM +Flag
SKOM +Gadd45
mmu-miR-3113
mmu-miR-429
mmu-miR-1196
mmu-miR-200b
mmu-miR-3098-5p
mmu-miR-378
mmu-miR-154*
mmu-miR-3472
mmu-miR-181c
mmu-miR-3470a
mmu-miR-324-3p
mmu-miR-205
mmu-miR-409-5p
mmu-miR-335-5p
mmu-miR-1892
mmu-miR-382*
mmu-miR-195
mmu-miR-3082-5p
mmu-miR-181d
mmu-miR-30c-2*
mmu-miR-320
mmu-miR-3069-3p
mmu-miR-676
mmu-miR-192
mmu-miR-434-5p
mmu-miR-134
mmu-miR-18a*
mmu-miR-700
mmu-miR-615-3p
mmu-miR-214*
mmu-miR-29b-1*
mmu-miR-31*
mmu-miR-483
mmu-miR-541
mmu-miR-337-3p
mmu-miR-223
mmu-miR-292-3p
mmu-miR-293
mmu-miR-22*
mmu-miR-132
mmu-miR-224
mmu-miR-302a
mmu-miR-294
mmu-miR-295
mmu-miR-291a-3p

**Figure S3**

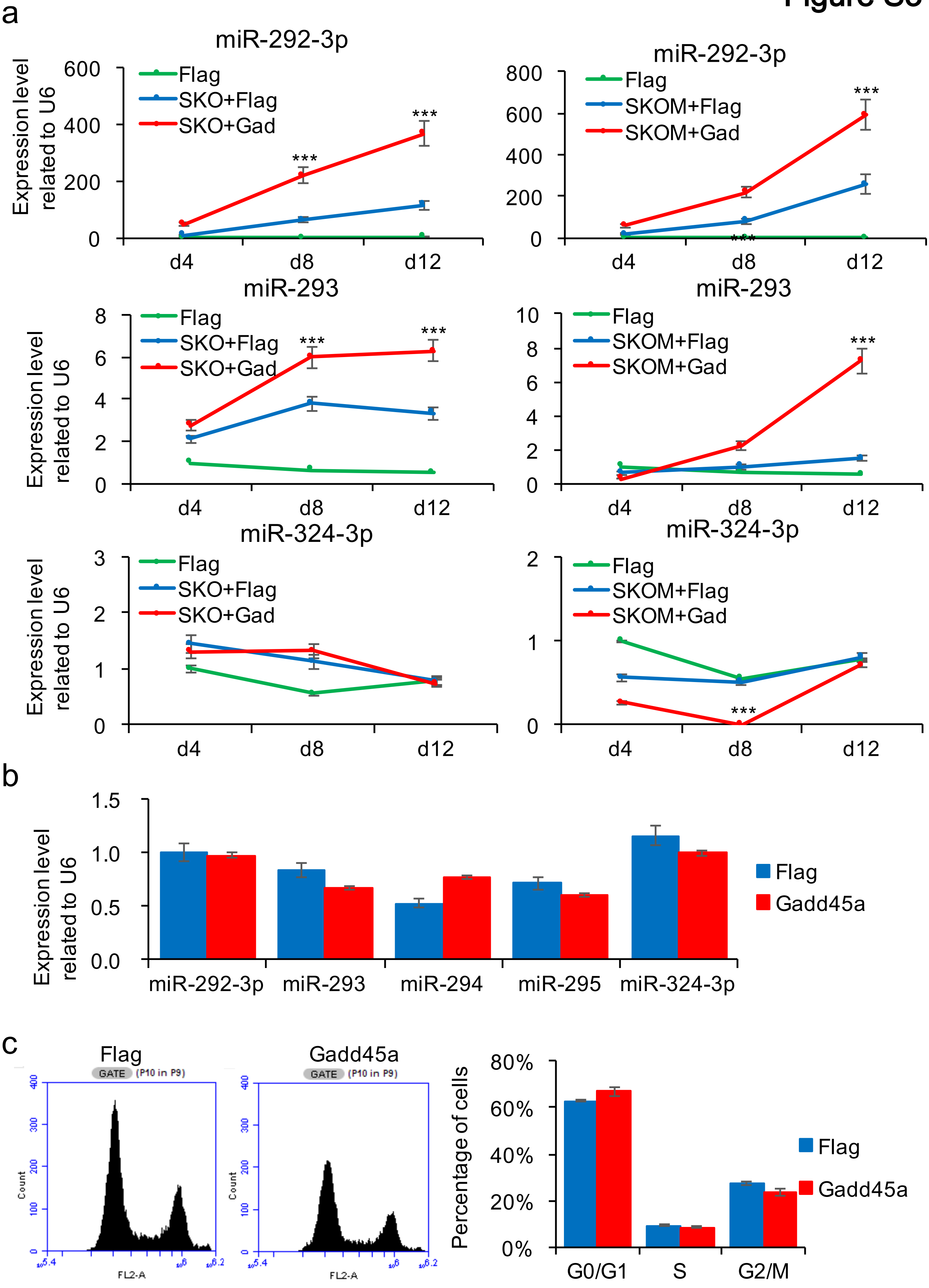
a
miR-292-3p
Expression level related to U6
600
400
200
0
Flag
SKO+Flag
SKO+Gad
***
***
d4
d8
d12
miR-292-3p
800
600
400
200
0
Flag
SKOM+Flag
SKOM+Gad
***
***
d4
d8
d12
miR-293
8
6
4
2
0
Flag
SKO+Flag
SKO+Gad
***
***
d4
d8
d12
miR-293
10
8
6
4
2
0
Flag
SKOM+Flag
SKOM+Gad
***
d4
d8
d12
miR-324-3p
3
2
1
0
Flag
SKO+Flag
SKO+Gad
d4
d8
d12
miR-324-3p
2
1
0
Flag
SKOM+Flag
SKOM+Gad
***
d4
d8
d12
b
1.5
1.0
0.5
0.0
miR-292-3p
miR-293
miR-294
miR-295
miR-324-3p
Flag
Gadd45a
c
Flag
GATE (P10 in P9)
Count
FL2-A
Gadd45a
GATE (P10 in P9)
Count
FL2-A
Percentage of cells
80%
60%
40%
20%
0%
G0/G1
S
G2/M
Flag
Gadd45a

# Figure S4

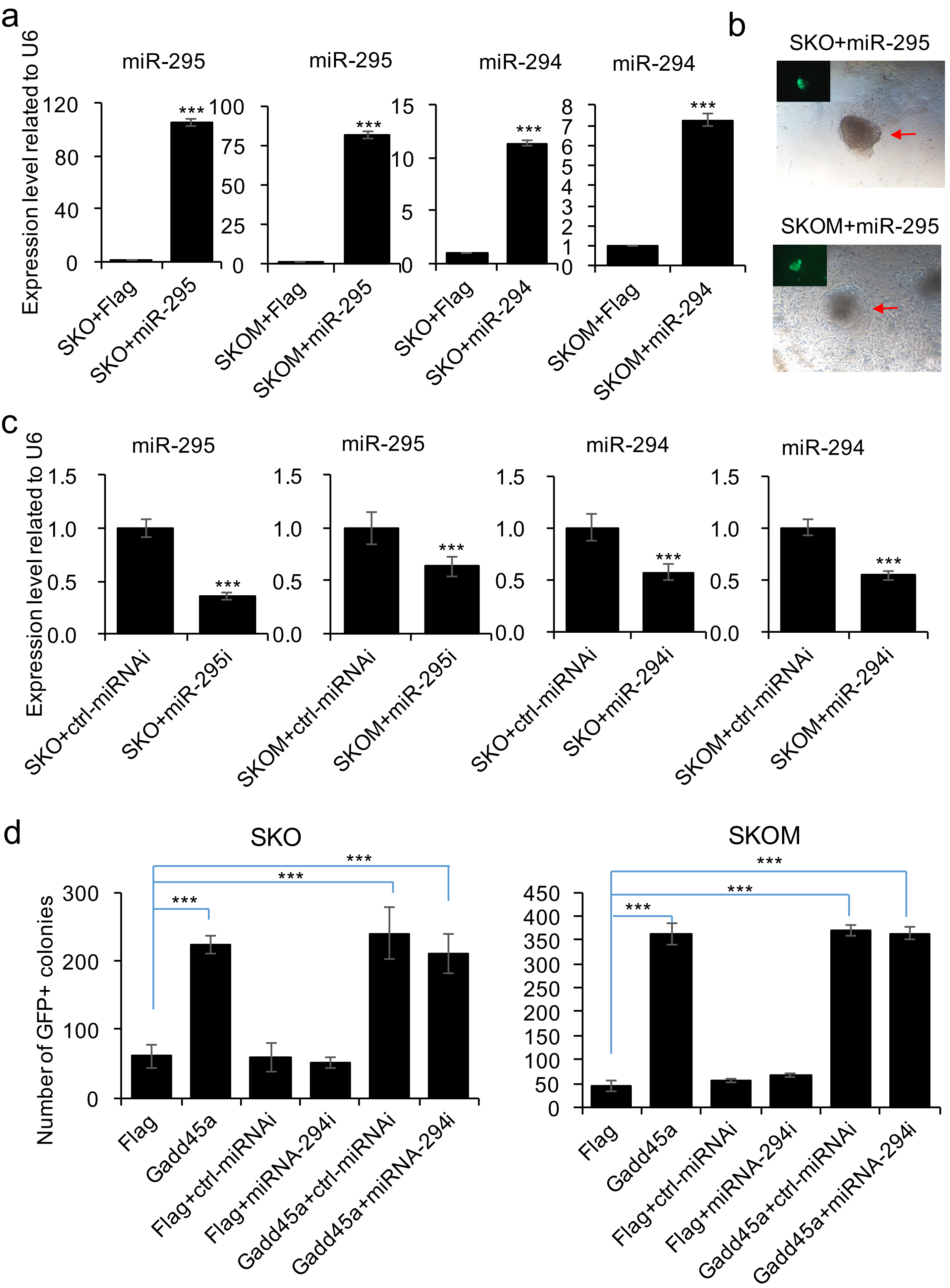

a
Expression level related to U6
miR-295
120
80
40
0
***
SKO+Flag
SKO+miR-295
miR-295
100
75
50
25
0
***
SKOM+Flag
SKOM+miR-295
miR-294
15
10
5
0
***
SKO+Flag
SKO+miR-294
miR-294
8
7
6
5
4
3
2
1
0
***
SKOM+Flag
SKOM+miR-294
b
SKO+miR-295
SKOM+miR-295
c
Expression level related to U6
miR-295
1.5
1.0
0.5
0.0
***
SKO+ctrl-miRNAi
SKO+miR-295i
miR-295
SKOM+ctrl-miRNAi
SKOM+miR-295i
miR-294
SKO+ctrl-miRNAi
SKO+miR-294i
miR-294
SKOM+ctrl-miRNAi
SKOM+miR-294i
d
SKO
Number of GFP+ colonies
300
200
100
0
***
Flag
Gadd45a
Flag+ctrl-miRNAi
Flag+miRNA-294i
Gadd45a+ctrl-miRNAi
Gadd45a+miRNA-294i
SKOM
450
400
350
300
250
200
150
100
50
0
***
Flag
Gadd45a
Flag+ctrl-miRNAi
Flag+miRNA-294i
Gadd45a+ctrl-miRNAi
Gadd45a+miRNA-294i

# Figure S5

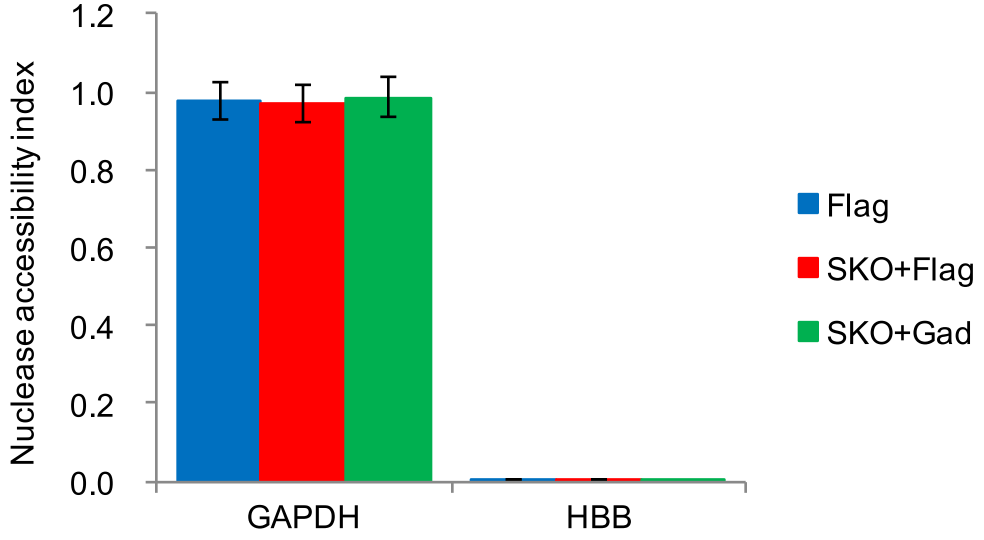

Nuclease accessibility index
1.2
1.0
0.8
0.6
0.4
0.2
0.0
GAPDH
HBB
Flag
SKO+Flag
SKO+Gad
